# Dietary index for gut microbiota score is inversely associated with carotid calcified plaque score in ischemic stroke patients

**DOI:** 10.3389/fnut.2026.1811243

**Published:** 2026-07-22

**Authors:** Yichao Guan, Dujuan Chang

**Affiliations:** 1 Department of Geriatrics, Central Hospital Affiliated to Shandong First Medical University, Jinan, China; 2 Department of Neurology, Central Hospital Affiliated to Shandong First Medical University, Jinan, China

**Keywords:** carotid artery calcification, dietary index for gut microbiota, gut microbiota, ischemic stroke, TMAO

## Abstract

**Background:**

Diet influences gut microbiota-derived metabolites, which may affect vascular inflammation and calcification. The Dietary Index for Gut Microbiota (DI-GM) captures dietary patterns hypothesized to support favorable microbial metabolite profiles. In this study, we examined whether higher DI-GM scores are associated with lower carotid calcification burden in ischemic stroke patients and whether trimethylamine N-oxide (TMAO) is statistically associated with this relationship, without implying causality.

**Method:**

In this cross-sectional study of 788 ischemic stroke patients from Central Hospital Affiliated to Shandong First Medical University, we calculated DI-GM scores from validated food frequency questionnaires and quantified carotid calcification using the Agatston method via computed tomography angiography. Plasma TMAO levels were measured by enzyme-linked immunosorbent assay (ELISA) ELISA.

**Results:**

Our findings revealed that participants in the highest DI-GM tertile had significantly lower calcification scores (76.07 ± 14.55) compared to the lowest tertile (316.00 ± 75.33, *p* < 0.001). Higher DI-GM scores correlated with lower TMAO, and decreased inflammatory markers (all *p* < 0.001). Each one-unit increase in DI-GM was independently associated with lower calcification odds (adjusted OR = 0.60, *p* = 0.02). Mediation analysis confirmed that TMAO significantly mediated these associations, accounting for substantial proportions of the total effects of DI-GM score on inflammatory markers, plaque thickness, and calcification score.

**Conclusion:**

Higher DI-GM score is cross-sectionally associated with lower carotid calcification and TMAO-mediated pathways, but causality remains unproven without longitudinal and microbiome-sequencing data.

## Introduction

Cerebrovascular accidents (CVA) remain a leading cause of mortality and disability worldwide (1). Carotid artery calcification, a marker of advanced atherosclerosis, is strongly associated with increased stroke risk and adverse cardiovascular outcomes (2). However, vascular calcification represents only one component of cerebrovascular risk biology and does not fully capture thrombosis-related mechanisms, endothelial dysfunction, or plaque instability, which are also critical determinants of stroke occurrence and progression (3). While traditional risk factors are well-established, emerging evidence suggests that dietary patterns and gut microbiota composition play critical roles in atherosclerotic disease pathogenesis (4).

The gut microbiota functions as a metabolically active organ influencing systemic inflammation, immune regulation, and metabolic homeostasis. Dysbiosis—characterized by reduced microbial diversity and elevated Firmicutes/Bacteroidetes (F/B) ratio—has been implicated in multiple cardiometabolic disorders (5, 6). Diet serves as a primary modulator of gut microbiota composition, with certain patterns promoting eubiosis and beneficial metabolite production, while others favor dysbiotic states and pro-inflammatory metabolite generation (7). Trimethylamine N-oxide (TMAO), produced through hepatic oxidation of gut bacteria-derived trimethylamine from dietary choline, phosphatidylcholine, and L-carnitine, has emerged as an important cardiovascular disease mediator (8). Elevated TMAO levels are associated with atherosclerotic plaque development and vascular calcification (9, 10). However, while TMAO-cardiovascular outcome relationships are established, the extent to which dietary patterns can modulate TMAO production and subsequently influence vascular calcification remains incompletely understood.

Short-chain fatty acids (SCFAs)—including butyrate, propionate, and acetate—represent another critical class of gut microbiota-derived metabolites with predominantly beneficial effects (11). Produced through bacterial fermentation of dietary fiber, SCFAs exert anti-inflammatory effects, improve intestinal barrier integrity, and modulate immune function (12). Fiber-rich, plant-based dietary patterns promote SCFA production, potentially counteracting pro-inflammatory and pro-atherogenic pathways (13).

Despite growing recognition of interconnections among diet, gut microbiota, inflammation, and cardiovascular disease, existing research has largely examined these components in isolation. Most dietary indices focus exclusively on traditional cardiovascular risk factors without incorporating gut microbiota-modulating properties or microbial metabolite production (14).

To address these gaps, we employed the Diet-Inflammation-Gut Microbiota (DI-GM) score—a novel composite dietary index integrating foods and nutrients that modulate gut microbiota composition, reduce systemic inflammation, and influence key microbial metabolites (15). The DI-GM score represents the first dietary assessment tool specifically designed to capture the complex interplay between dietary intake, gut microbiota ecology, inflammatory pathways, and metabolite production (16). While the association between established dietary indices like the Dietary Inflammatory Index (DII) and carotid plaque vulnerability has been studied in patients with ischemic stroke (17, 18), the effect of the new DI-GM score on carotid plaque vulnerability has not yet been investigated.

The novelty of this study is multifold. First, this is the first investigation to examine the DI-GM index in relation to clinical outcomes, specifically carotid artery calcification and stroke severity. Second, while TMAO has been studied extensively as a cardiovascular risk marker, we uniquely examine how a comprehensive dietary pattern—rather than isolated nutrients—influences TMAO production and subsequently affects vascular calcification. Third, we investigate the dual mediating roles of microbial metabolites (TMAO) in pathways linking dietary scores to inflammatory biomarkers, vascular calcification, and neurological outcomes. Fourth, we employ mediation analysis to partition diet effects on calcification into direct pathways and indirect pathways operating through microbiota composition and metabolite production, providing preliminary evidence consistent with microbiota-related metabolic pathways linking dietary patterns and vascular disease.

We hypothesized that higher DI-GM scores would be associated with: (1) favorable metabolite profiles (lower TMAO); (2) lower systemic inflammation; (3) lower carotid calcification burden; and (4) less severe neurological deficits. Furthermore, we hypothesized that associations between DI-GM scores and clinical outcomes would be substantially mediated through alterations in metabolite production. These findings have the potential to inform dietary recommendations for future risk reduction in stroke patients, provide evidence for microbiota-targeted nutritional interventions, and advance understanding of mechanistic pathways linking diet, gut microbiota, and cerebrovascular disease.

## Methods

### Study design and participants

This cross-sectional study was conducted at the Department of Neurology, Central Hospital Affiliated to Shandong First Medical University (Jinan, Shandong Province, China). Patients presenting with acute ischemic stroke were consecutively recruited from the neurology department. The study protocol was approved by the Medical Research Ethics Committee of Central Hospital Affiliated to Shandong First Medical University (Protocol No. 20251105006), and all participants provided written informed consent prior to enrollment. Inclusion criteria were: (1) age ≥18 years; (2) confirmed diagnosis of acute ischemic stroke based on clinical presentation and neuroimaging (computed tomography or magnetic resonance imaging); (3) admission within 48 h of symptom onset; and (4) ability to provide informed consent or availability of a legally authorized representative. Exclusion criteria included: (1) use of antibiotics or probiotics within the preceding 3 months; (2) inflammatory bowel disease, celiac disease, or other chronic gastrointestinal disorders; (3) active malignancy; (4) recent gastrointestinal surgery (within 6 months); (5) severe hepatic or renal dysfunction (alanine aminotransferase >3 × upper limit of normal, estimated glomerular filtration rate <30 mL/min/1.73 m^2^); (6) immunosuppressive therapy; and (7) incomplete dietary or clinical data. These criteria were implemented to minimize confounding factors that could independently influence gut microbiota composition (e.g., antibiotics, probiotics, chronic gastrointestinal diseases), systemic inflammation (e.g., active malignancy, immunosuppressive therapy), or metabolite production (e.g., severe hepatic/renal dysfunction), (8) presence of severe dysphagia requiring enteral tube feeding at the time of enrollment; (9) malnutrition defined as NRS-2002 score ≥3 thereby allowing a clearer assessment of the association between habitual dietary patterns and the outcomes of interest.

### Sample size calculation

**Figure 1:**
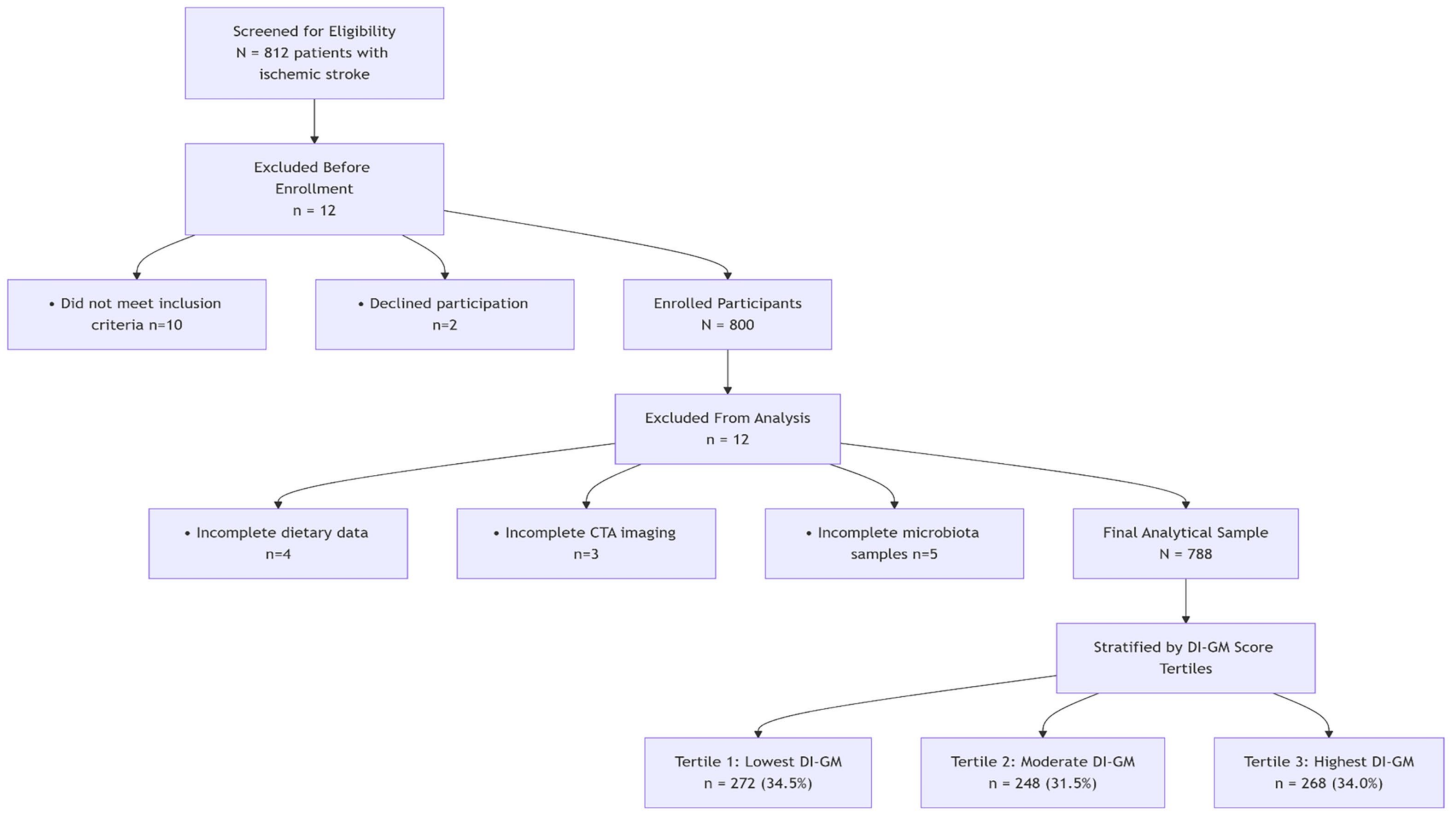
Participant flow diagram. Flow of participants through the study. Of adults with acute ischemic stroke initially recruited, 12 were excluded due to incomplete dietary (*n* = 4), imaging (*n* = 3), or microbiota data (*n* = 5). The final analytical sample comprised 788 participants.

Sample size was calculated based on an expected moderate effect size (Cohen’s *f*^2^ = 0.15) for the association between DI-GM score and carotid calcification score, with *α* = 0.05 (two-sided) and power = 0.80. Using multiple linear regression with up to 8 covariates, the required sample size was calculated as approximately 650 participants using GPower software (version 3.1). To account for potential non-normally distributed outcomes, the use of multiple regression models, subgroup analyses across DI-GM tertiles, and anticipated missing data or exclusions (estimated at 10–15%), we aimed to recruit a minimum of 800 participants. This sample size provides adequate statistical power for the primary analysis and sufficient precision for stratified analyses and mediation modeling. A total of 800 adults with ischemic stroke were initially recruited, of whom 788 met all eligibility criteria and had complete data for the primary analyses, yielding a final analytical sample that exceeded the minimum required sample size (see Figure 1 flowchart).

### Clinical and anthropometric assessments

Demographic information, medical history, and lifestyle factors were collected through standardized questionnaires administered by trained research personnel. Information included age, sex, smoking status (current, former, never), alcohol consumption (yes/no), physical activity (yes/no), history of hypertension, diabetes mellitus, hyperlipidemia, and atrial fibrillation. Use of statins, antiplatelet agents, and antihypertensive medications (as a single category) was ascertained through patient interview and review of electronic medical records, and was recorded as binary variables (yes/no). Detailed information on specific drug classes, dosages, and treatment duration was not consistently available across the cohort. Body weight was measured to the nearest 0.1 kg using a calibrated digital scale with participants wearing light clothing and no shoes. Height was measured to the nearest 0.1 cm using a wall-mounted stadiometer. Body mass index (BMI) was calculated as weight (kg) divided by height squared (m^2^). Neurological deficit severity was assessed using the National Institutes of Health Stroke Scale (NIHSS) at admission by certified neurologists. The NIHSS is a 15-item standardized assessment tool with scores ranging from 0 (no deficit) to 42 (severe deficit) (19).

### Dietary assessment and DI-GM score calculation

Dietary intake was assessed using a validated, semi-quantitative food frequency questionnaire (FFQ) comprising 110 food items with standard portion sizes. Because acute ischemic stroke may impair swallowing function and nutritional intake, proxy-assisted dietary reporting was used when appropriate. The FFQ was designed to capture habitual dietary intake during the 12 months preceding stroke onset rather than acute post-stroke dietary changes. To minimize potential recall bias in the acute stroke setting, several measures were implemented: (1) the FFQ focused on habitual intake over the prior 12 months, reducing the influence of recent per stroke dietary alterations; (2) patients with severe dysphagia requiring enteral tube feeding were excluded; (3) dietary information was corroborated using proxy reports from family caregivers and validation against medical records; and (4) all participants underwent Nutritional Risk Screening 2002 (NRS-2002), and those with malnutrition (NRS-2002 score ≥3) were excluded from primary analyses. The FFQ captured the frequency of consumption (e.g., times per day, week, or month) and usual portion size (e.g., bowl, cup, or grams) for each item. Daily intake in grams for specific food groups (e.g., vegetables, fruits, nuts, meat, dairy) was calculated by summing the gram intake of all individual items within each group. For instance, total vegetable intake was derived by aggregating intakes of all vegetable items listed in the FFQ, such as leafy greens, root vegetables, and others. Nutrient intakes, including dietary fiber, were calculated by multiplying the gram intake of each food item by its nutrient composition per gram based on the Chinese Food Composition Table and summing across all items. Daily intakes of nutrients and food groups were computed using the Chinese Food Composition Table and Nutritionist IV software (version 7.0; N-Squared Computing), as previously described in the validation study for this FFQ (20). Energy-adjusted dietary intakes (g/1,000 kcal/day) were calculated by dividing each dietary component by total daily energy intake (kcal) and multiplying by 1,000.

The Dietary Index for Gut Microbiota (DI-GM) was calculated according to the method of Kase et al. (15), which includes 14 components: 10 beneficial items (fermented dairy products, chickpeas, soy products, whole grains, dietary fiber, cranberries, avocados, broccoli, coffee, and green tea) and four adverse items (red meat, processed meats, refined grains, and a high fat diet [≥40% of energy from fat]). Because DI-GM scoring is based on sex-specific median intake, it is less dependent on absolute energy intake; however, total energy intake was included in multivariable models to minimize residual confounding. This index was originally developed and validated against measures of gut microbiota composition (e.g., Shannon diversity index) and metabolite production (e.g., short-chain fatty acids), demonstrating that higher DI-GM scores correlate with a more favorable gut microbial profile (21). For each component, sex-specific median intake levels were derived from the study population; beneficial components scored 1 point if intake was at or above the median (otherwise 0), while adverse components scored 1 point if intake was below the median (otherwise 0). The points were summed to yield a total DI-GM score ranging from 0 to 14, with higher scores indicating greater scores to a gut-microbiota-beneficial dietary pattern. The continuous score was used in regression analyses, and participants were also categorized into tertiles (T1–T3) based on the score distribution for descriptive comparisons.

### Carotid artery imaging and calcification assessment

Carotid artery calcification was objectively confirmed using contrast-enhanced computed tomography angiography (CTA), which served as the primary imaging modality for quantitative vascular calcification assessment. Carotid computed tomography angiography (CTA) was performed using a dual-source 64-slice CT system (Siemens Healthcare, Erlangen, Germany). Participants were positioned supine with the neck slightly extended. Imaging was acquired by scanning from 4 cm below the aortic arch to the superior border of the orbit in the craniocaudal direction. Standard imaging parameters were applied according to manufacturer protocols. Carotid artery calcification scores were measured using the Syngo Calcium Scoring system (Siemens Healthcare, Germany). Artery calcification was defined as a focus of ≥4 contiguous pixels accompanied by a CT density of ≥130 Hounsfield units (HU). According to the Agatston method, calcification was divided into 4 density grades based on peak CT attenuation: 130–199 HU (Grade 1), 200–299 HU (Grade 2), 300–399 HU (Grade 3), and ≥400 HU (grade 4). The total Agatston calcification score was calculated as the sum of each individual lesion’s score, which equals the calcification area (mm^2^) multiplied by the corresponding maximal density coefficient (1–4). Bilateral carotid arteries were analyzed, and the total calcification score represented the sum of all calcified lesions in both carotid arterial systems. To ensure methodological validity and reproducibility, all CTA measurements and calcification scoring procedures were independently performed by two experienced radiologists who were blinded to participants’ dietary intake, DI-GM scores, inflammatory biomarkers, and clinical characteristics. All CT measurements and calcification scoring were performed independently by two experienced radiologists with more than 10 years of cardiovascular imaging expertise, both blinded to participants’ clinical characteristics, dietary information, and DI-GM scores. Inter-observer reliability was assessed using intraclass correlation coefficients (ICC), which exceeded 0.90 for calcification scores. Discrepancies between the two readers were resolved through consensus review and discussion. In addition to quantitative calcification scoring, calcification severity was graded according to a standardized qualitative classification: Grade 1 (no calcification or minimal calcification with maximum thickness <2 mm), Grade 2 (moderate calcification with thickness 2–4 mm or involving <50% of plaque area), and Grade 3 (severe calcification with thickness >4 mm or involving ≥50% of plaque area) (17, 22).

### Laboratory analyses

All blood samples were collected in the morning after an overnight fast, with patients instructed to take their regular morning medications after the blood draw to avoid acute interference from oral medications on the measured parameters. Fasting venous blood samples (approximately 20 mL) were collected in the morning (07:00–09:00) after an overnight fast of at least 8 h. Samples were collected in tubes containing EDTA, serum separator, or sodium heparin as appropriate. Serum and plasma were separated by centrifugation at 3,000 × g for 10 min at 4 °C and stored at −80 °C until analysis. Standard biochemical parameters were measured using automated analyzers. Fasting blood glucose, lipid profile (total cholesterol, LDL-cholesterol, HDL-cholesterol, triglycerides), uric acid, and creatinine were measured using a standard enzymatic colorimetric method on a Cobas c702 analyzer (Roche Diagnostics). Estimated glomerular filtration rate (eGFR) was calculated using the CKD-EPI equation. Fibrinogen was measured by the Clauss method. C-reactive protein (CRP) was measured by high-sensitivity immunoturbidimetric assay (inter-assay coefficient of variation <5%). Interleukin-6 (IL-6) and tumor necrosis factor-alpha (TNF-α) were measured by enzyme-linked immunosorbent assay (ELISA) using commercial kits (R&D Systems, Minneapolis, MN, USA) according to manufacturer protocols. Erythrocyte sedimentation rate (ESR) was measured by the Westergren method. Neutrophil-to-lymphocyte ratio (NLR) was calculated from complete blood count differential.

Plasma trimethylamine N-oxide (TMAO) concentrations were measured using a commercially available quantitative competitive ELISA kit (Human Trimethylamine-N-oxide ELISA Kit, MyBioSource, MBS7269386, San Diego, CA, USA) following the manufacturer’s instructions. All samples were analyzed in duplicate, with intra-assay and inter-assay coefficients of variation <10% and <12%, respectively.

### Statistical analysis

Missing data (<2% for key variables) were handled via complete case analysis. Participants with missing data on key variables (dietary intake, calcification measurements, or microbiota data) were excluded from relevant analyses. All statistical analyses were performed using Stata/SE version 14.0 (StataCorp, College Station, TX, USA). Continuous variables were tested for normality using the Shapiro–Wilk test. Differences between participants with and without carotid calcification were assessed using independent *t*-tests for normally distributed continuous variables, Mann–Whitney *U* tests for non-normally distributed variables, and chi-square tests for categorical variables. Participants were stratified into tertiles based on DI-GM score distribution (T1: lowest scores, T2: moderate scores, T3: highest scores). Differences across tertiles were examined using one-way analysis of variance (ANOVA) with Bonferroni *post-hoc* tests for normally distributed continuous variables. Linear regression was used to examine associations between DI-GM score (independent variable) and continuous outcomes including inflammatory biomarkers, TMAO, NIHSS score, maximum plaque thickness, and calcification score. Three models were constructed: Model 1, unadjusted; Model 2, adjusted for age, sex, and BMI; Model 3, further adjusted for total energy intake (kcal/day), physical activity, and alcohol consumption. Given the lack of detailed, complete medication data and potential for over adjustment bias, pharmacological variables were not included in the multivariable models. Regression coefficients (*β*) with 95% confidence intervals (CI) are reported. Logistic regression was performed to assess the association between DI-GM score and the binary outcome of calcification presence (yes/no). Structural equation modeling (SEM) was employed to test whether the associations between DI-GM score and outcomes (inflammatory biomarkers, plaque thickness, calcification score, NIHSS score) were associated with microbial metabolites (TMAO). The product-of-coefficients method was used to calculate indirect effects. Bootstrapping with 5,000 resamples was used to generate bias-corrected 95% confidence intervals for indirect effects. The total effect was partitioned into direct effect (association between DI-GM score and outcome after accounting for the mediator) and indirect effect (association between DI-GM score and outcome operating through the mediator). Due to the cross-sectional design, we cannot establish temporal precedence between dietary exposure, metabolite measurements, and calcification outcomes. This is particularly relevant given the temporal mismatch between chronic calcification burden (reflecting cumulative pathology over years) and single-time-point measurements of dynamic metabolites such as TMAO. While TMAO exhibits moderate intra-individual stability under stable dietary conditions, and our DI-GM score assessed 12-month habitual intake, reverse causality and unmeasured confounding cannot be excluded. Therefore, our mediation analyses should be interpreted as hypothesis-generating rather than causal. Prospective studies with repeated metabolite measurements and longitudinal calcification assessment are required to establish temporal sequence.

## Results

A total of 788 patients with cerebrovascular accident (CVA) were included in the analysis. The mean age of participants was approximately 69 years, and 55% were male. Carotid calcification was identified in 404 participants (51.3%), while 384 (48.7%) had no detectable calcification.

### Comparison by calcification status

As presented in Table 1, participants with carotid calcification exhibited significantly higher BMI and markedly elevated inflammatory biomarkers, including NLR, CRP, IL-6, TNF-α, and ESR (all *p* < 0.001). Indicators of gut dysbiosis, including an elevated TMAO levels, were also more pronounced in the calcification group (*p* < 0.001). The use of statins, antihypertensive agents, and antiplatelet medications did not differ significantly between participants with and without carotid calcification (all *p* > 0.05).

**Table 1:** Comparison of clinical, inflammatory, microbiota, and metabolite variables according to carotid calcification status.

Variable	No calcification (*n* = 384) mean ± SD	Calcification (*n* = 404) mean ± SD	*p*-value
BMI (kg/m^2^)	26.39 ± 1.52	27.25 ± 1.63	<0.001
Medication use, *n* (%)			
Statin use	330 (85.9)	348 (86.1)	0.935
Antihypertensive use	334 (87.0)	356 (88.1)	0.628
Antiplatelet use	309 (80.5)	332 (82.2)	0.538
DI-GM score	9.64 ± 1.31	6.95 ± 1.39	<0.001
NLR	2.45 ± 0.66	3.27 ± 0.94	<0.001
CRP (mg/dL)	3.79 ± 1.23	6.85 ± 1.96	<0.001
IL-6 (pg/mL)	4.29 ± 1.29	7.48 ± 2.11	<0.001
TNF-α (pg/mL)	2.40 ± 0.85	4.30 ± 1.62	<0.001
ESR (mm/h)	22.43 ± 9.39	32.93 ± 11.70	<0.001
TMAO (μmol/L)	5.14 ± 1.08	7.42 ± 2.07	<0.001

Data presented as mean ± SD. Between-group comparisons performed using independent *t*-tests for normally distributed variables and Mann–Whitney *U* tests for non-normally distributed variables. BMI, body mass index; NLR, neutrophil-to-lymphocyte ratio; CRP, C-reactive protein; IL-6, interleukin-6; TNF-α, tumor necrosis factor-alpha; ESR, erythrocyte sedimentation rate; TMAO, trimethylamine N-oxide.

Notably, the DI-GM score was substantially lower in participants with calcification (6.95 ± 1.39) compared with those without calcification (9.64 ± 1.31, *p* < 0.001).

### Baseline characteristics across DI-GM tertiles

Table 2 summarizes participant characteristics according to tertiles of DI-GM scores. No statistically significant differences were observed across tertiles for age, sex, smoking status, hypertension, atrial fibrillation, blood pressure, lipid profile, or fasting blood glucose. However, BMI differed significantly, with lower values observed in the highest DI-GM tertile (*p* < 0.001). Medication use (statins, antihypertensives, antiplatelets) did not differ significantly across DI-GM tertiles (all *p* > 0.05).

**Table 2:** Baseline characteristics of participants according to DI-GM tertiles (*n* = 788).

Variable	T1 (lowest DI-GM) (*n* = 272, range: 0–7)	T2 (*n* = 248, range: 7–9)	T3 (highest DI-GM) (*n* = 268, range: 10–14)	*p*-value
Demographic				
Age (years)	69.68 ± 5.63	69.05 ± 6.42	69.48 ± 4.27	0.415
Sex (male)	142 (52.2%)	136 (54.8%)	158 (59.0%)	0.284
Smoking status	92 (33.8%)	90 (36.3%)	91 (34.0%)	0.805
HTN	177 (65.1%)	158 (63.7%)	175 (65.3%)	0.921
Alcohol consumption, *n* (%)	34 (12.5)	31 (12.5)	33 (12.3)	0.99
AF	24 (8.8%)	16 (6.5%)	27 (10.1%)	0.321
BMI (kg/m^2^)	27.13 ± 1.46	27.39 ± 1.83	26.02 ± 1.24	<0.001
Medication use, *n* (%)				
Statin use	238 (87.5)	214 (86.3)	226 (84.3)	0.612
Antihypertensive use	240 (88.2)	219 (88.3)	231 (86.2)	0.735
Antiplatelet use	222 (81.6)	201 (81.0)	218 (81.3)	0.982
Dietary factors				
Energy (kcal/d)	2,217 ± 188	2,312 ± 179	2063 ± 181	<0.001
Protein (g/d)	62.24 ± 6.71	69.89 ± 7.59	63.38 ± 4.47	<0.001
Carbohydrate (g/d)	324.66 ± 21.44	327.48 ± 22.46	292.35 ± 13.92	<0.001
Total fat (g/d)	74.40 ± 8.80	80.33 ± 7.54	71.12 ± 12.71	<0.001
Fiber (g/d)	20.69 ± 2.88	26.92 ± 4.13	29.93 ± 8.87	<0.001
Vegetables (g/d)	226.24 ± 27.84	282.15 ± 39.33	304.40 ± 89.26	<0.001
Fruits (g/d)	132.60 ± 16.73	153.98 ± 19.92	167.28 ± 38.59	<0.001
Nuts (g/d)	9.16 ± 3.27	12.76 ± 4.48	10.00 ± 3.03	<0.001
Meat (g/d)	67.13 ± 11.56	78.18 ± 11.68	70.25 ± 11.92	<0.001
Dairy (g/d)	15.87 ± 2.81	17.94 ± 3.20	20.51 ± 6.31	<0.001
Dietary factors (energy-adjusted)				
Protein (g/1,000 kcal)	28.1 ± 3.0	30.2 ± 3.3	30.7 ± 2.2	<0.001
Fat (g/1,000 kcal)	33.6 ± 4.0	34.7 ± 3.3	34.5 ± 6.2	<0.001
Fiber (g/1,000 kcal)	9.33 ± 1.30	11.64 ± 1.79	14.51 ± 4.30	<0.001
Vegetables (g/1,000 kcal)	102.1 ± 12.6	122.0 ± 17.0	147.5 ± 43.2	<0.001
Fruits (g/1,000 kcal)	59.8 ± 7.5	66.6 ± 8.6	81.1 ± 18.7	<0.001
Nuts (g/1,000 kcal)	4.13 ± 1.47	5.52 ± 1.94	4.85 ± 1.47	<0.001
Meat (g/1,000 kcal)	30.3 ± 5.2	33.8 ± 5.1	34.0 ± 5.8	<0.001
Dairy (g/1,000 kcal)	7.16 ± 1.27	7.76 ± 1.38	9.94 ± 3.06	<0.001
Inflammatory biomarkers				
NLR	3.19 ± 0.73	3.35 ± 1.04	2.11 ± 0.18	<0.001
CRP (mg/dL)	6.94 ± 1.65	6.07 ± 1.97	3.09 ± 0.54	<0.001
IL-6 (pg/mL)	7.63 ± 1.77	6.62 ± 1.97	3.55 ± 0.89	<0.001
TNF-α (pg/mL)	4.35 ± 1.35	3.86 ± 1.57	1.92 ± 0.47	<0.001
ESR (mm/h)	32.26 ± 9.81	34.33 ± 12.27	17.28 ± 2.64	<0.001
Microbiota indices				
TMAO (μmol/L)	7.84 ± 1.79	6.36 ± 1.74	4.69 ± 0.93	<0.001
Cardiometabolic risk factors				
Uric acid	6.73 ± 0.29	6.78 ± 0.37	6.26 ± 0.25	<0.001
Fibrinogen	3.59 ± 0.31	3.65 ± 0.25	3.31 ± 0.31	<0.001
Systolic BP	128.90 ± 8.19	130.02 ± 8.01	129.48 ± 8.53	0.303
Diastolic BP	81.99 ± 4.18	82.34 ± 4.26	82.75 ± 4.33	0.116
FBS	101.34 ± 4.23	102.24 ± 4.56	101.84 ± 4.56	0.068
HDL-C	40.96 ± 6.98	39.77 ± 6.94	40.54 ± 7.26	0.158
LDL-C	117.54 ± 6.77	118.31 ± 6.84	118.16 ± 7.34	0.411
Triglycerides	129.44 ± 5.08	130.53 ± 5.21	130.72 ± 5.05	0.188
Total cholesterol	186.91 ± 11.94	188.19 ± 11.95	188.88 ± 12.47	0.161
eGFR	86.40 ± 8.80	88.33 ± 7.54	88.12 ± 12.71	0.052

*p*-value for trend across tertiles assessed by one-way ANOVA with Bonferroni *post-hoc* test for normally distributed variables.

Medication use variables are presented as *n* (%). The denominators for each medication row are the total number of participants with available data for that specific medication. Not all patients were prescribed each medication, and medication categories are not mutually exclusive; therefore, the sum across rows does not equal the total sample size. No significant differences in medication use were observed across DI-GM tertiles (all *p* > 0.05).

HTN, hypertension; AF, atrial fibrillation; FBS, fasting blood glucose; HDL-C, high-density lipoprotein cholesterol; LDL-C, low-density lipoprotein cholesterol; eGFR, estimated glomerular filtration rate. Energy adjusted values were calculated as mean of individual ratios (g/1,000 kcal).

Dietary intake variables demonstrated clear gradients across tertiles. Participants in the highest tertile reported higher intakes of fiber, vegetables, fruits, nuts, and dairy products, along with lower total energy, carbohydrate, and fat intake (all *p* < 0.001). Analyses using energy-adjusted dietary variables yielded similar trends across DI-GM tertiles.

A consistent pattern was observed for inflammatory and microbiota-related variables. Higher DI-GM scores was associated with lower NLR, CRP, IL-6, TNF-α, ESR, TMAO, uric acid, and fibrinogen levels.

### Calcification burden and stroke severity across DI-GM tertiles

Marked differences in calcification burden were observed across DI-GM tertiles (Table 3). Calcification presence was observed in 95.6% of participants in the lowest tertile (T1), compared with 48.4% in T2 and 9.0% in the highest tertile (T3) (*p* < 0.001).

**Table 3:** Calcification burden and stroke severity across DI-GM tertiles.

Variable	DI-GM Tertiles 1 (*n* = 272)	DI-GM Tertiles 2 (*n* = 248)	DI-GM Tertiles 3 (*n* = 268)	*p*-value
Calcification presence, *n* (%):				<0.001
No calcification	12 (4.4%)	128 (51.6%)	244 (91.0%)	
Calcification present	260 (95.6%)	120 (48.4%)	24 (9.0%)	
Calcification grade, *n* (%):				<0.001
Grade 1	12 (4.4%)	136 (54.8%)	244 (91.0%)	
Grade 2	32 (11.8%)	68 (27.4%)	12 (4.5%)	
Grade 3	228 (83.8%)	44 (17.7%)	12 (4.5%)	
Maximum plaque thickness (mm)	4.77 ± 1.24	4.13 ± 1.98	2.06 ± 1.19	<0.001
Calcification score	316.00 ± 75.33	197.00 ± 125.89	76.07 ± 14.55	<0.001
NIHSS score	7.97 ± 2.66	5.39 ± 2.30	3.3 ± 1.83	<0.001

Calcification grade: Grade 1 = no/minimal calcification (<2 mm thickness); Grade 2 = moderate calcification (2–4 mm or <50% plaque area); Grade 3 = severe calcification (>4 mm or ≥50% plaque area). NIHSS, National Institutes of Health Stroke Scale.

*p*-values derived from chi-square test for categorical variables and ANOVA for continuous variables.

Similarly, severe calcification (Grade 3) was predominantly concentrated in T1 (83.8%) and was infrequent in T3 (4.5%). Mean maximum plaque thickness, Agatston calcification score, and NIHSS score progressively decreased across increasing DI-GM tertiles (all *p* < 0.001).

Mean maximum plaque thickness demonstrated a progressive decline across increasing DI-GM tertiles (4.77 ± 1.24 mm in T1, 4.13 ± 1.98 mm in T2, and 2.06 ± 1.19 mm in T3; *p* < 0.001). The Agatston calcification score followed a comparable pattern, with the highest values in T1 (316.00 ± 75.33) and the lowest in T3 (76.07 ± 14.55) (*p* < 0.001). Stroke severity, assessed using the NIHSS score, also decreased markedly across tertiles (7.97 ± 2.66 in T1 vs. 1.98 ± 0.83 in T3; *p* < 0.001).

### Linear regression analysis

As shown in Table 4, higher DI-GM scores were significantly associated with lower levels of CRP, IL-6, TNF-α, TMAO, NIHSS score, plaque thickness, and calcification score in unadjusted and adjusted models. In the fully adjusted model, each one-unit increase in DI-GM score was associated with lower levels of CRP (*β* = −1.22), IL-6 (*β* = −1.14), TNF-α (*β* = −1.25), and TMAO (*β* = −1.30) (all *p* < 0.001). Higher DI-GM scores were also associated with lower NIHSS score (*β* = −0.45), lower maximum plaque thickness (*β* = −1.13 mm), and lower calcification score (*β* = −0.014) (all *p* < 0.001). These associations remained statistically significant across unadjusted and partially adjusted models, indicating consistency of the observed relationships.

**Table 4:** Association of DI-GM with inflammatory, clinical, and calcification markers in patients with CVA.

Variable	Model 1a: B (95% CI)	*p*-value	Model 2b: B (95% CI)	*p*-value	Model 3c: B (95% CI)	*p*-value
CRP (mg/dL)	−0.63 (−0.675, −0.595)	<0.001	−0.95 (−0.995, −0.909)	<0.001	−1.22 (−1.29, −1.183)	<0.001
IL-6 (pg/mL)	−0.61 (−0.650, −0.576)	<0.001	−0.79 (−0.838, −0.759)	<0.001	−1.14 (−1.85, −1.091)	<0.001
TNF-α (pg/mL)	−0.79 (−0.857, −0.734)	<0.001	−1.25 (−1.324, −1.176)	<0.001	−1.25 (−1.33, −1.176)	<0.001
TMAO (μmol/L)	−0.65 (−0.70, −0.603)	<0.001	−0.96 (−1.017, −0.907)	<0.001	−1.30 (−1.37, −1.227)	<0.001
NIHSS score	−0.34 (−0.373, −0.323)	<0.001	−0.45 (−0.481, −0.422)	<0.001	−0.45 (−0.50, −0.424)	<0.001
Maximum plaque thickness (mm)	−0.58 (−0.627, −0.511)	<0.001	−1.21 (−1.31, −1.128)	<0.001	−1.13 (−1.22, −1.04)	<0.001
Calcification score (Agatston)	−0.011 (−0.012, −0.010)	<0.001	−0.0145 (−0.0152, −0.0138)	<0.001	−0.013 (−0.015, −0.013)	<0.001

*p*-values based on linear regression.

Model 1a: Unadjusted. Model 2b: Adjusted for age, sex, and BMI. Model 3: adjusted for Model 2 + total energy intake, physical activity (yes/no), alcohol consumption (yes/no).

### Logistic regression for calcification presence

Table 5 presents the results of logistic regression analyses examining the association between DI-GM score and the binary outcome of calcification presence. In the crude model, each one-unit increase in DI-GM score was associated with 69% lower odds of having carotid calcification (OR = 0.31, 95% CI: 0.27–0.36, *p* < 0.001). This association strengthened after adjustment for age, sex, and body mass index, with each unit increase in DI-GM score associated with 79% lower odds of calcification (OR = 0.21, 95% CI: 0.17–0.27, *p* < 0.001).

**Table 5:** Logistic regression analysis for the association between DI-GM score and calcification presence.

Predictor	Crude OR (95% CI)	*p*-value	Adjusted OR^†^ (95% CI)	*p*-value	Fully adjusted OR^‡^ (95% CI)	*p*-value
DI-GM score	0.31 (0.27–0.36)	<0.001	0.21 (0.17–0.27)	<0.001	0.60 (0.39–0.93)	0.02
Sex (Female vs. Male)	—	—	0.92 (0.88–0.97)	<0.001	0.92 (0.88–0.97)	<0.001
Age (years)	—	—	1.48 (1.22–1.79)	<0.001	1.36 (1.10–1.68)	0.005
BMI (kg/m^2^)	—	—	1.68 (1.37–2.06)	<0.001	3.27 (2.09–5.09)	<0.001
CRP	—	—	—	—	1.55 (1.41–2.46)	<0.001

Outcome: Calcification presence (Yes vs. No).

^†^Adjusted for sex, age, BMI, physical activity, and alcohol consumption. ^‡^Additionally adjusted for NLR and CRP.

In the fully adjusted model additionally controlling for neutrophil-to-lymphocyte ratio and C-reactive protein, the DI-GM score retained an independent association with calcification presence, though the magnitude was attenuated (OR = 0.60, 95% CI: 0.39–0.93, *p* = 0.02). This attenuation suggests that inflammatory pathways partially account for the association between dietary scores and calcification risk.

### Mediation analysis through TMAO

**Figure 2:**
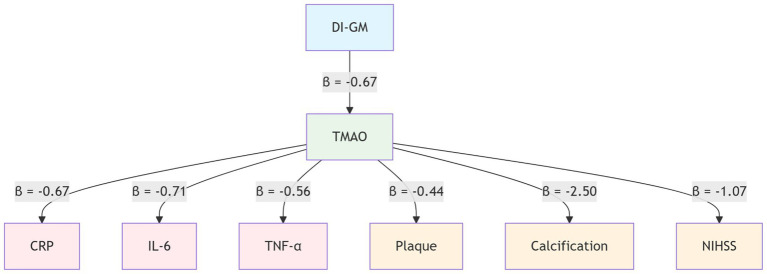
Mediation pathways linking DI-GM score to carotid calcification. Structural equation modeling showing direct and indirect effects of the Dietary Index for Gut Microbiota (DI-GM) on carotid calcification burden. Solid arrows indicate statistically significant paths (all *p* < 0.05). Values represent unstandardized path coefficients (*β*). CRP, C-reactive protein; IL-6, interleukin-6; TNF-α, tumor necrosis factor-alpha; NIHSS, National Institutes of Health Stroke Scale.

Mediation analysis (Table 6 and Figure 2) demonstrated that the association between DI-GM score and inflammatory, and neurological markers was statistically partitioned into direct and indirect components through TMAO. Mediation analysis demonstrated that trimethylamine N-oxide (TMAO) significantly mediated the associations between DI-GM score and all clinical outcomes. Higher DI-GM scores was strongly associated with lower TMAO levels (*β* = −0.67, 95% CI: −0.73 to −0.61, *p* < 0.001). The indirect effects of DI-GM score through TMAO were significant for all inflammatory markers, vascular outcomes, and neurological severity. For inflammatory markers, the indirect effect on CRP was *β* = −0.67 (95% CI: −0.73 to −0.61), on IL-6 was *β* = −0.71 (95% CI: −0.77 to −0.65), and on TNF-α was *β* = −0.56 (95% CI: −0.60 to −0.52); the direct effect on TNF-α was not significant (*β* = −0.01, *p* > 0.05), indicating near-complete mediation. For vascular outcomes, the indirect effect on maximum plaque thickness was *β* = −0.44 (95% CI: −0.49 to −0.39) and on carotid calcification score was *β* = −2.50 (95% CI: −3.62 to −2.38). For neurological severity, the indirect effect on NIHSS score was *β* = −1.07 (95% CI: −1.17 to −0.98). All indirect pathways were statistically significant (*p* < 0.001), demonstrating that TMAO may represent a biologically relevant intermediary biomarker between gut microbiota-targeted dietary patterns and reduced inflammation, vascular calcification, and stroke severity.

**Table 6:** Mediation analysis of the association between DI-GM and calcification markers, inflammatory, and neurological outcomes through TMAO.

Outcome variable	Path	Effect type	*β* (95% CI)
DI-GM score → TMAO			
		Total	−1.39
CRP	DI-GM score → CRP	Direct	−0.20 (−0.24, −0.17)
	DI-GM score → TMAO → CRP	Indirect	−0.67 (−0.73, −0.61)
		Total	−0.87
IL-6	DI-GM score → IL-6	Direct	−0.24 (−0.27, −0.21)
	DI-GM score → TMAO → IL-6	Indirect	−0.71 (−0.77, −0.65)
		Total	−0.95
TNF-α	DI-GM score → TNF-α	Direct	−0.01 (NS)
	DI-GM SCORE → TMAO → TNF-α	Indirect	−0.56 (−0.60, −0.52)
		Total	−0.57
Maximum plaque thickness (mm)	DI-GM score → maximum plaque thickness	Direct	−0.12 (−0.19, −0.06)
	DI-GM score → TMAO → maximum plaque thickness	Indirect	−0.44 (−0.49, −0.39)
		Total	−0.56
Calcification score (Agatston)	DI-GM score → calcification score	Direct	−2.12 (−2.23, −2.20)
	DI-GM score → TMAO → calcification score	Indirect	−2.50 (−3.62, −2.38)
		Total	−5.62
NIHSS score	DI-GM score → NIHSS	Direct	−0.32 (−0.41, −0.24)
	DI-GM score → TMAO → NIHSS	Indirect	−1.07 (−1.17, −0.98)
		Total	−1.38

*β* coefficients represent unstandardized path estimates derived from structural equation modeling using maximum likelihood estimation (Stata/SE 18.0).

Indirect effects were calculated using the product-of-coefficients method.

All paths were statistically significant at *p* < 0.05 unless marked NS. Negative coefficients indicate that higher DI-GM score is associated with lower levels of inflammatory markers, vascular plaque burden, calcification score, and neurological severity. Analyses were based on 780 participants with complete data.

## Discussion

The present study investigated the interrelationships between DI-GM scores and gut microbial characteristics, systemic inflammation, carotid calcification burden, and neurological severity among patients with ischemic stroke. Importantly, the DI-GM score has previously demonstrated correlations with gut microbial *α*-diversity and the Firmicutes/Bacteroidetes ratio (15, 21); however, in the present study we did not conduct direct 16S rRNA sequencing and instead measured microbial metabolites (TMAO) as functional proxies of gut microbiota activity. Our findings support the hypothesis that higher DI-GM scores were consistently associated with lower TMAO concentrations, lower inflammatory biomarkers, reduced calcification burden, and lower NIHSS scores. These associations persisted across crude and adjusted models and were supported by mediation analyses demonstrating indirect pathways through TMAO, positioning diet, microbiota, microbial metabolites, inflammation, and neurological severity within a coherent biological axis.

Participants in the highest DI-GM tertile exhibited significantly lower inflammatory markers and TMAO concentrations compared with the lowest tertile. Elevated TMAO is implicated in atherosclerosis, vascular calcification, and cardiovascular events, whereas SCFAs promote endothelial function and mitigate inflammation (10). Prior studies largely examined individual dietary components or metabolites (23), whereas the present study applied a comprehensive dietary index specifically targeting gut microbiota. TMAO is produced via hepatic flavin-containing monooxygenase 3 (FMO3) conversion of trimethylamine, derived from gut microbial metabolism of dietary choline, phosphatidylcholine, and L-carnitine, promoting calcification through macrophage cholesterol accumulation, endothelial dysfunction, platelet hyperreactivity, and osteogenic differentiation of vascular smooth muscle cells (9, 10). The findings extend prior research on dietary inflammatory potential (24). While studies using the DII associated pro-inflammatory diets with elevated inflammatory biomarkers, they did not incorporate microbiota-specific dietary components or microbial metabolites (14). Here, DI-GM scores showed substantial mediation through microbiota-related pathways (55–77% of associations with inflammatory markers), highlighting the mechanistic role of gut microbiota. SCFAs exert anti-inflammatory effects via histone deacetylase inhibition, NF-κB suppression, G-protein coupled receptor activation, and enhancement of gut barrier integrity, collectively reducing systemic exposure to inflammatory bacterial products (12, 25). Concurrently, lower TMAO concentrations attenuate NLRP3 inflammasome activation and macrophage-driven inflammatory responses (26). While Mediterranean and MIND diets have been linked to beneficial microbial profiles in general populations, prior studies in stroke patients have not applied indices explicitly designed to target gut microbiota metabolites. In this study, TMAO mediated 50% of the association between DI-GM score and maximum plaque thickness, demonstrating that dietary effects on vascular calcification operate substantially through microbial metabolite pathways. Diets enriched in fermentable fiber, polyphenols, and fermented foods promote beneficial gut microbiota composition and anti-inflammatory metabolite production, whereas diets high in animal protein and saturated fat are associated with increased TMAO production, systemic inflammation, and metabolic dysfunction (27). Importantly, direct gut microbiome sequencing was not performed in this study. Therefore, although TMAO are widely regarded as microbiota-associated metabolites, the present analyses cannot definitively establish that the observed associations were mediated specifically by alterations in microbial taxa or microbial diversity. Alternative explanations, including broader cardiometabolic, inflammatory, or nutritional mechanisms independent of gut microbiota composition, remain possible. Consequently, the findings should be interpreted as indirect evidence consistent with microbiota-related pathways rather than direct confirmation of microbiome-mediated causality.

Calcification burden decreased across DI-GM tertiles. Prior studies linked dietary inflammatory potential or specific nutrients to vascular calcification but did not consider microbiota-mediated pathways (17, 18). Here, TMAO mediated a significant proportion of the diet–calcification association. Pro-inflammatory cytokines (IL-1β, IL-6, and TNF-α) directly promote vascular calcification through endothelial-to-mesenchymal transition, activation of bone morphogenetic protein 2, and upregulation of calcification enzymes (28). SCFAs counteract these processes via anti-inflammatory signaling, endothelial support, and modulation of smooth muscle phenotype, while reduced TMAO limits oxidative stress and macrophage-mediated calcification (29). The cross-sectional design precludes causal inference regarding diet–calcification relationships. Reverse causality remains possible, as stroke severity may influence dietary patterns through dysphagia or functional impairment. Prospective cohort studies and randomized controlled trials are required to establish temporal precedence and test causality.

NIHSS scores progressively decreased with higher DI-GM scores, with TMAO serving as mediators. Altered gut microbiota, elevated TMAO, and systemic inflammation exacerbate stroke severity by influencing neuroinflammation, blood–brain barrier integrity, cerebrovascular reactivity, and collateral circulation (30). SCFAs provide neuroprotective effects, whereas TMAO promotes oxidative stress and neuroinflammatory activation, demonstrating a gut–brain–vascular link (31, 32).

The clinical utility of microbiota-oriented dietary assessment should also be considered in the context of economic feasibility. Although advanced metabolomic analyses and CTA imaging are costly and may not be widely accessible, the DI-GM score is derived from conventional dietary assessment tools and may therefore represent a relatively low-cost approach for identifying dietary patterns associated with vascular risk. In the present study, imaging and metabolomic analyses were primarily used to explore biological plausibility rather than as routine screening tools. Clinically, higher DI-GM scores were associated with lower inflammation, reduced carotid calcification burden, and less severe neurological impairment. However, because carotid calcification reflects a chronic cumulative process whereas TMAO and SCFA levels are relatively dynamic, the mediation analyses should be interpreted cautiously as cross-sectional statistical associations rather than definitive evidence of longitudinal causal mediation.

The interpretation of gut microbiota-related findings in clinical populations must consider the substantial impact of commonly prescribed medications. Beyond dietary patterns, drugs such as statins, metformin, proton pump inhibitors, and antiplatelet agents are known to modulate gut microbiota composition and metabolite levels (21). In the present study, the majority of participants were receiving secondary prevention therapies, including statins and antiplatelet agents. However, because detailed medication data (specific agents, dosages, and duration) were not fully available, and because these therapies are tightly linked to the very cardiometabolic conditions that are diet-related, we did not include medication variables in our primary multivariable analyses. Nonetheless, we cannot rule out residual confounding from unmeasured aspects of pharmacotherapy, and future work should incorporate comprehensive medication profiles.

The present study did not include detailed thrombosis-related vascular biomarkers such as D-dimer, platelet activation indices, coagulation factor measurements, endothelial function markers, or advanced plaque vulnerability imaging. Therefore, although carotid calcification is a recognized marker of chronic atherosclerotic burden, the current findings should not be interpreted as direct evidence for stroke prevention efficacy. Rather, the observed associations suggest that higher DI-GM adherence is linked to a more favorable vascular and inflammatory profile among patients with ischemic stroke. An additional consideration is the potential influence of dysphagia and stroke-related malnutrition on dietary assessment. Although the FFQ was designed to capture long-term habitual dietary intake (the 12 months preceding stroke onset), severe neurological deficits may still affect either the accuracy of dietary recall or the patient’s pre-stroke nutritional status. Moreover, because formal swallowing assessments and standardized malnutrition screening tools were not systematically collected in all participants, we cannot entirely exclude residual bias related to reverse causality—for instance, the possibility that unrecognized pre-stroke malnutrition or subclinical dysphagia influenced dietary patterns or metabolite profiles independently of the DI-GM score. Future prospective studies should incorporate objective swallowing evaluations and serial nutritional assessments to address this limitation.

Methodological considerations include the cross-sectional design, which limits causal inference; reliance on food frequency questionnaires subject to recall bias; potential residual confounding; single-center population; and not assessment 16S rRNA-based microbiota analysis. Dietary assessment reflected the prior 12 months, whereas vascular calcification is a chronic process. While we adjusted for potential confounders the observational design cannot entirely rule out residual confounding from unmeasured factors or the precise impact of specific drug regimens. Further, medication data were limited to broad categories (use/no use) without information on specific agents, doses, or adherence, which may confound the observed associations and should be addressed in future studies. Furthermore, while our study demonstrates associations between DI-GM score and carotid calcification—a marker of advanced atherosclerosis—we did not directly assess thrombosis-related parameters (e.g., platelet function, fibrinogen activity, D-dimer, or tissue factor expression). Emerging evidence suggests that TMAO directly enhances platelet reactivity and thrombosis risk (33, 34); thus, the mediating role of TMAO in thrombosis pathways warrants dedicated investigation in future studies. Given that acute ischemic stroke frequently results from thromboembolic events superimposed on atherosclerotic plaques, future studies should incorporate comprehensive thrombotic biomarkers to fully evaluate the potential of DI-GM-guided interventions for stroke prevention. Future research should include prospective cohorts, randomized trials, metagenomic analyses to identify microbial taxa and functional pathways, and exploration of gene–diet–microbiota interactions. Combined interventions incorporating prebiotics, probiotics, or synbiotics alongside dietary modulation warrant further investigation.

## Conclusion

In conclusion, higher scores of DI-GM is associated with favorable metabolite profiles, lower systemic inflammation, minor carotid calcification, and lower neurological severity in ischemic stroke patients, substantially mediated through gut microbiota metabolites. These findings underscore the diet–microbiota–vascular–neurological axis and provide a foundation for future research on microbiota-targeted dietary interventions to mitigate cerebrovascular disease risk.

## Data Availability

The raw data supporting the conclusions of this article will be made available by the authors, without undue reservation.
